# Associations between trans fatty acids and systemic immune-inflammation index: a cross-sectional study

**DOI:** 10.1186/s12944-024-02109-w

**Published:** 2024-04-27

**Authors:** Xiao-Feng Zhu, Yu-Qi Hu, Zhi-Cheng Dai, Xiu-Juan Li, Jing Zhang

**Affiliations:** 1 Department of Clinical Medicine, The Nanshan College of Guangzhou Medical University, Guangzhou, 511436 China; 2 Department of Clinical Medicine, The Third Clinical School of Guangzhou Medical University, Guangzhou, 511436 China; 3 Department of Orthopedics, Shanghai General Hospital, Shanghai Jiao Tong University School of Medicine, Shanghai, 201600 China; 4 Second Department of Infectious Disease, Shanghai Fifth People’s Hospital, Fudan University, Shanghai, 201100 China

**Keywords:** Systemic immunity inflammation index, Cross-sectional study, National health and nutrition examination survey, Trans fatty acids

## Abstract

**Background:**

Previous studies have demonstrated that trans fatty acids (TFAs) intake was linked to an increased risk of chronic diseases. As a novel systemic inflammatory biomarker, the clinical value and efficacy of the systemic immune-inflammation index (SII) have been widely explored. However, the association between TFAs and SII is still unclear. Therefore, the study aims to investigate the connection between TFAs and SII in US adults.

**Methods:**

The study retrieved data from the National Health and Nutrition Examination Survey (NHANES) for the years 1999–2000 and 2009–2010. Following the exclusion of ineligible participants, the study encompassed a total of 3047 individuals. The research employed a multivariate linear regression model to investigate the connection between circulating TFAs and SII. Furthermore, the restricted cubic spline (RCS) model was utilized to evaluate the potential nonlinear association. Subgroup analysis was also conducted to investigate the latent interactive factors.

**Results:**

In this investigation, participants exhibited a mean age of 47.40 years, with 53.91% of them being female. Utilizing a multivariate linear regression model, the independent positive associations between the log2-transformed palmitelaidic acid, the log2 transformed-vaccenic acid, the log2-transformed elaidic acid, the log2-transformed linolelaidic acid, and the log2-transformed-total sum of TFAs with the SII (all *P* < 0.05) were noted. In the RCS analysis, no nonlinear relationship was observed between the log2-transformed palmitelaidic acid, the log2 transformed-vaccenic acid, the log2-transformed elaidic acid, the log2-transformed linolelaidic acid, the log2-transformed-total sum of TFAs and the SII (all *P* for nonlinear > 0.05). For the stratified analysis, the relationship between the circulating TFAs and the SII differed by the obesity status and the smoking status.

**Conclusions:**

A positive association was investigated between three types of TFA, the sum of TFAs, and the SII in the US population. Additional rigorously designed studies are needed to verify the results and explore the potential mechanism.

**Supplementary Information:**

The online version contains supplementary material available at 10.1186/s12944-024-02109-w.

## Introduction

Trans fatty acids (TFAs) are a specific type of unsaturated acids that are naturally occurring and artificially produced. In the U.S., dietary TFAs account for 2–3% of the energy intake, primarily from processed foods, including baked products and packaged snacks [1]. However, TFAs are not essential to the human body and are detrimental to health. Earlier investigations have established that the intake of TFAs is associated with an increase in lipid levels [2, 3], which may lead to an increased prevalence of cardiovascular diseases [4]. Moreover, studies based on in vivo and in vitro models found that the TFAs could not only modulate the microbiome in the mice but also induce inflammation and oxidative stress [5, 6], which are associated with the risk of some common chronic diseases [7].

It has been proposed that inflammation is a major factor in the development of diseases. To better evaluate the systematic inflammation of patients in clinical practice, a novel blood inflammation biomarker called the systematic immune-inflammation index (SII) has been proposed, which could be calculated based on three types of blood cells (lymphocytes, neutrophils, and platelets) [8]. As an easily accessible indicator, plenty of studies have investigated and confirmed its prognostic value in diabetes, lung cancer, and the general population [9–11]. A study based on 6003 Chinese adults discovered that the SII was significantly associated with hypertension over a long-term period [12]. In addition, recent studies have found that elevated SII may increase the risk of diabetic retinopathy and cognitive impairment, as well as the severity of carotid artery stenosis [13–15].

Some studies have reported that a few dietary factors, including dietary fiber, vitamin D and selenium, may influence systemic inflammation in humans [16–18]. However, information on the association between TFAs and systemic inflammation is limited. Given the widespread use of TFAs and the excellent efficacy of SII, exploring the relationship between circulating TFAs and SII may provide some novel insights into the adverse effects of TFAs on inflammation. Hence, National Health and Nutrition Examination Survey (NHANES) data collected during the years 1999–2000 and 2009–2010 were used in the study to explore the connections between plasma TFAs and SII among U.S. adults.

## Methods

### Study population

NHANES is a large database that could be freely accessed by researchers around the globe. The Centers for Disease Control and Prevention (CDC) conducted the NHANES project on a two-year cycle to evaluate the nutritional and medical status of non-institutionalized individuals living in the U.S. Approximately 5000 civilians living in the communities were selected by authorities across each cycle. The complex sampling and multi-stage methodology was utilized in the sample survey to generate nationally representative data.

**Fig. 1:**
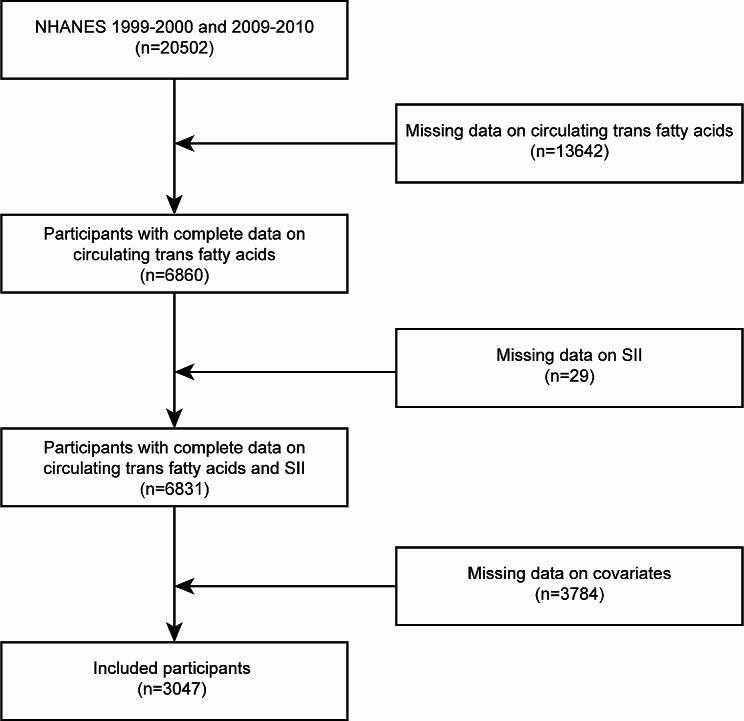
Flow chart of participant selection. Abbreviations: NHANES, National Health and Nutrition Examination Survey, SII, Systemic immune-inflammation index

The research selected participants’ data from two survey cycles of the database (1999–2000 and 2009–2010), for which the level of circulating TFAs was available. In this study, a total of 20,502 participants aged ≥ 20 years were first extracted. Then, we excluded 13,642 samples with missing data on TFAs in the second step and 29 samples with missing data on SII in the third step. Furthermore, 3784 participants with missing data on the covariates were also regarded as ineligible. Finally, 3047 eligible U.S. adults from the NHANES were included to conduct a cross-sectional study. The flowchart of the inclusion and exclusion criteria is shown in Fig. 1. The protocol was approved by the Ethical Review Committee of the National Health Council, and each individual gave written informed consent.

### Measurement of circulating TFA

Previous studies have reported detailed methods and approaches to evaluate the level of plasma TFA [19, 20]. In brief, participants’ blood samples were obtained in the morning after a fasting period following the protocol outlined by the CDC. Subsequently, TFA isomers were identified by their chromatographic retention times and specific mass-to-charge ratios. Quantification of metabolites was conducted using established standard solutions, incorporating stable isotope-labeled fatty acids as internal standards. The total amount of TFAs was determined as follows: Sum TFAs = vaccenic acid + linoelaidic acid + palmitelaidic acid + elaidic acid.

### Identification of SII

The study derived the SII by multiplying the number of neutrophils by the number of platelets, followed by dividing by the number of lymphocytes. The level of the complete blood cell count is expressed as ×103 cells/µl and was assessed by blood analysis equipment, which is conducted by professional laboratory staff.

### Covariates

Considering the clinical facts, the potential confounding factors were included in the study. Demographic factors, including age, gender, race, education, poverty income ratio (PIR), and marital status, were evaluated through a questionnaire conducted at the mobile examination center. Race was categorized into five groups: Mexican American, non-Hispanic Black, non-Hispanic White, other Hispanic, and other races. Marital status was categorized as married/living with a partner, widowed/divorced/separated, or never married. Smoking status was defined based on lifetime cigarette consumption, with categories for never smoked, ever smoked, and current smoker. Alcohol consumption was determined by the mean alcohol intake over a two-day diet obtained through dietary recall. Education level was stratified into three groups: less than high school, high school graduate, and more than high school. Trained medical personnel measured and calculated participants’ body mass index (BMI) during interviews. Information on cardiovascular disease (CVD), hypertension, cancer, and diabetes mellitus (DM) was collected through questionnaires. Specifically speaking, participants were considered CVD patients, based on the previous studies [21–23]. The direct immunoassay-related equipment was utilized for examining the level of the lipids in individuals. Serum uric acid levels were measured using the colorimetric method in laboratory tests, and the estimated glomerular filtration rate (eGFR) was calculated following established research protocols [24].

### Statistical analysis

Based on the CDC guideline, all analyses involved in the study took clustering, multi-stage, and sample weights into consideration. Given the skewed distribution of TFAs, a log2 transformation was applied for the regression analysis. The baseline characteristics of participants were stratified by the tertiles of sum TFAs. Continuous variables were presented as mean ± standard error using weighted linear regression models, while categorical variables were expressed as percentages through the Rao-Scott chi-square test. Subsequently, the research employed the multivariate linear regression model to examine the relationship between TFAs and SII. The effect size (β) and 95% confidence intervals (CI) were calculated for statistical assessment. Model 1 was unadjusted, while Model 2 accounted for age, gender, and race. Model 3 was adjusted for the all latent confounders we included for the present investigation to verify the robustness of the results. Additionally, the restricted cubic spline (RCS) model was utilized to investigate potential non-linear associations involving four main types of TFAs, the sum TFAs, and SII. Furthermore, subgroup analysis and interactive *P* values were utilized to probe potential interaction effects among stratified variables. All analyses were conducted using R software (version 4.2.1).

## Results

### Baseline characteristics of the study participants

Table 1 presents the weighted basic characteristics of 3047 individuals. In the study population, the average age was 47.40 years, and 53.91% were female. Additionally, the mean levels of the circulating palmitelaidic acid, vaccenic acid, elaidic acid and linolelaidic acid were 5.05 µmol/L, 25.87 µmol/L, 20.99 µmol/L, and 2.07 µmol/L, respectively. After classifying by sum TFAs tertiles, individuals with higher circulating TFAs were more likely to be older, non-Hispanic White, have lower educational attainment, married/living with a partner, current smokers, less alcohol consumption, lower eGFR, and higher SII. However, no statistically significant difference was shown in gender, PIR, uric acid, CVD, hypertension, DM, and cancer across the three groups. Interestingly, BMI was shown to be highest in the T2 group with an average of 29.24 kg/m2 and the population in the T2 group had the highest age with an average of 48.49 years.

**Table 1:** Demographics and characteristics of participants from NHANES 1999–2000 and 2009-2010

Variable	Sum TFAS (µmol/L)				
	Overall	T1	T2	T3	P value
Age (years)	47.40 ± 0.53	45.90 ± 0.81	48.49 ± 0.65	48.29 ± 0.99	0.022
Gender (%)					0.764
Male	46.09	46.69	46.39	44.79	
Female	53.91	53.31	53.61	55.21	
Race (%)					<0.001
Mexican American	8.16	7.59	9.60	7.21	
Non-Hispanic Black	9.37	11.47	8.98	6.70	
Non-Hispanic White	72.02	66.76	72.50	79.31	
Other Hispanic	5.21	5.29	5.95	4.17	
Other Race	5.24	8.89	2.97	2.61	
Education Level (%)					<0.001
Less Than High School	18.07	14.28	18.69	22.99	
High School Graduate	23.55	18.46	24.91	29.48	
More Than High School	58.38	67.26	56.40	47.53	
Marital Status (%)					<0.001
Married/Living with a partner	66.41	64.51	66.27	69.42	
Widowed/Divorced/Separated	17.80	15.23	20.89	17.81	
Never Married	15.79	20.27	12.84	12.76	
Smoking Status (%)					0.043
Never	54.42	58.40	54.84	47.91	
Former	26.86	26.16	26.48	28.40	
Current	18.72	15.44	18.68	23.69	
Alcohol consumption (g/d)	7.74 ± 0.49	10.78 ± 0.75	7.40 ± 0.90	3.58 ± 0.34	<0.001
PIR	3.01 ± 0.05	3.12 ± 0.05	2.99 ± 0.07	2.89 ± 0.11	0.092
BMI (kg/m^2^)	28.56 ± 0.19	27.98 ± 0.31	29.24 ± 0.26	28.58 ±0.31	0.023
Uric acid (mg/dl)	0.43 ± 0.04	5.35 ± 0.06	5.52 ± 0.07	5.44 ± 0.05	0.075
eGFR (ml/min/1.73 m^2^)	94.27 ± 0.65	97.31 ± 0.99	92.61 ± 0.83	91.77 ± 1.04	<0.001
HDL (mg/dl)	53.70 ± 0.49	57.49 ± 0.57	53.39 ± 0.60	48.37 ± 0.59	<0.001
LDL (mg/dl)	119.24 ± 1.18	109.68 ± 1.85	121.99 ± 1.88	130.14 ± 1.43	<0.001
CVD (%)					0.174
Yes	8.41	7.34	8.93	9.35	
No	91.59	92.66	91.07	90.65	
Hypertension (%)					0.325
Yes	29.58	27.49	31.52	30.30	
No	70.42	72.51	68.48	69.70	
DM (%)					0.431
Yes	7.15	7.12	8.10	6.02	
No	92.85	92.88	91.90	93.98	
Cancer (%)					0.061
Yes	9.90	8.27	11.37	10.52	
No	90.10	91.73	88.63	89.48	
Palmitelaidic acid (µmol/L)	5.05 ± 0.11	3.11 ± 0.04	4.94 ± 0.05	8.12 ± 0.15	<0.001
Vaccenic acid (µmol/L)	25.87 ± 0.72	13.49 ± 0.14	24.03 ± 0.16	46.74 ± 1.02	<0.001
Elaidic acid (µmol/L)	20.99 ± 0.72	9.82 ± 0.15	18.73 ± 0.14	40.55 ± 1.16	<0.001
Linolelaidic acid (µmol/L)	2.07 ± 0.06	1.30 ± 0.02	1.97 ± 0.04	3.35 ± 0.10	<0.001
SII	539.57 ± 7.48	504.15 ± 10.48	533.91 ± 13.62	599.82 ± 16.27	<0.001

Mean ± SD for continuous variables: the P value was calculated by the weighted linear regression model

(%) for categorical variables: the P value was calculated by the weighted chi-square test

Abbreviations: TFAs, trans fatty acids, BMI, Body mass index, PIR, Poverty income ratio, eGFR, Estimated glomerular filtration rate, CVD, Cardiovascular disease, DM, Diabetes mellitus, SII, Systemic immune-inflammation index, HDL, high-density lipoprotein, LDL, low-density lipoprotein

### Relationship between TFAs and SII

The multivariate linear regression model was performed and detailed results were shown in Table 2. In the crude model (model 1), the four types of TFA and the sum of TFAs were significantly and positively related to SII. After adjusting for age, sex, and race (model 2), the relationship was weakened. After adjusting for the covariates that were included in the study in Model 3, the connection between the log2-transformed palmitelaidic acid (β = 56.84, 95% CI = 30.93, 82.74, *P* < 0.001), the log2-transformed vaccenic acid (β = 32.28, 95% CI = 14.99, 49.57, *P* = 0.002), the log2-transformed elaidic acid (β = 40.31, 95% CI = 23.09, 57.54, *P* < 0.001), the log2-transformed-linolelaidic acid (β = 27.04, 95% CI = 6.10, 47.97, *P* = 0.016), the log2-transformed sum TFAs (β = 40.33, 95% CI = 21.29, 59.38, *P* < 0.001) and SII remain robust. Compared to the T1 group, individuals in the T3 group of palmitelaidic acid (β = 75.19, 95% CI = 25.38, 125.00, *P* = 0.007), vaccenic acid (β = 62.02, 95% CI = 11.02, 113.02, *P* = 0.022), elaidic acid (β = 84.43, 95% CI = 34.80, 134.07, *P* = 0.003), and sum TFAs (β = 78.08, 95% CI = 31.74, 124.41, *P* = 0.003) were significantly had higher SII. However, the population in the T3 group of the linolelaidic acid was not observed to have a higher SII (*P* > 0.05).

**Table 2:** The associations of circulating trans fatty acids with SII

Exposure	Model 1 β (95%Cl) P value	Model 2 β (95%Cl) P value	Model 3 β (95%Cl) P value
Palmitelaidic acid (µmol/L)	63.10 (42.04, 84.17) <0.001	59.24 (39.26, 79.22) <0.001	56.84 (30.93, 82.74) <0.001
Palmitelaidic acid tertile			
T1	Reference	Reference	Reference
T2	4.29 (-29.66, 38.24) 0.797	0.07 (-34.90, 35.04) 0.997	-6.37 (-44.24, 31.50) 0.718
T3	94.84 (53.70,135.98) <0.001	87.69 (48.14, 127.24) <0.001	75.19 (25.38, 125.00) 0.007
P for trend	<0.001	<0.001	0.007
Vaccenic acid (µmol/L)	39.29 (23.48, 55.10) <0.001	39.05 (24.13, 53.96) <0.001	32.28 (14.99, 49.57) 0.002
Vaccenic acid tertile			
T1	Reference	Reference	Reference
T2	7.48 (-20.77, 35.73) 0.592	5.53 (-22.76, 33.82) 0.691	-0.41 (-31.04, 30.22) 0.977
T3	75.13 (28.43, 121.83) 0.003	74.23 (30.37, 118.08) 0.002	62.02 (11.02, 113.02) 0.022
P for trend	0.003	0.002	0.023
Elaidic acid (µmol/L)	51.70 (35.01, 68.39) <0.001	49.16 (33.56, 64.76) <0.001	40.31 (23.09, 57.54) <0.001
Elaidic acid tertile			
T1	Reference	Reference	Reference
T2	47.29 (8.96, 85.62) 0.017	43.61 (4.58, 82.63) 0.030	31.52 (-0.53, 63.57) 0.053
T3	109.64 (64.96, 154.32) <0.001	103.4 (60.40, 146.39) <0.001	84.43 (34.80, 134.07) 0.003
P for trend	<0.001	<0.001	0.003
Linolelaidic acid (µmol/L)	41.72 (22.03, 61.41) <0.001	39.00 (20.97, 57.02) <0.001	27.04 (6.10, 47.97) 0.016
Linolelaidic acid tertile			
T1	Reference	Reference	Reference
T2	30.58 (-24.12, 85.27) 0.262	28.59 (-24.55, 81.72) 0.278	17.48(-38.55, 73.51) 0.506
T3	55.74 (19.45, 92.03) 0.004	51.27 (17.02, 85.53) 0.005	30.44( -5.19, 66.07) 0.087
P for trend	0.005	0.006	0.094
Sum TFAs (µmol/L)	50.25 (32.68, 67.82) <0.001	48.37 (32.00, 64.75) <0.001	40.33 (21.29, 59.38) <0.001
Sum TFAs tertile			
T1	Reference	Reference	Reference
T2	29.00 (-9.26, 67.25) 0.132	26.26 (-11.46, 63.98) 0.164	16.91 (-16.89, 50.71) 0.294
T3	95.34 (53.12, 137.55) <0.001	92.00 (52.17, 131.83) <0.001	78.08 (31.74, 124.41) 0.003
P for trend	<0.001	<0.001	0.003

Model 1: no covariates were adjusted

Mode 2: age, gender, and race were adjusted

Mode 3: age, gender, race, PIR, education level, BMI, marital, smoking status, CVD, alcohol consumption, uric acid, eGFR, HDL, LDL, hypertension, DM, and cancer

Abbreviations: SII, Systemic immune-inflammation index, PIR, Poverty income ratio, BMI, Body mass index, CVD, Cardiovascular disease, eGFR, Estimated glomerular filtration rate, HDL, high-density lipoprotein, LDL, low-density lipoprotein, DM, Diabetes mellitus, CI, Confidence interval

**Fig. 2:**
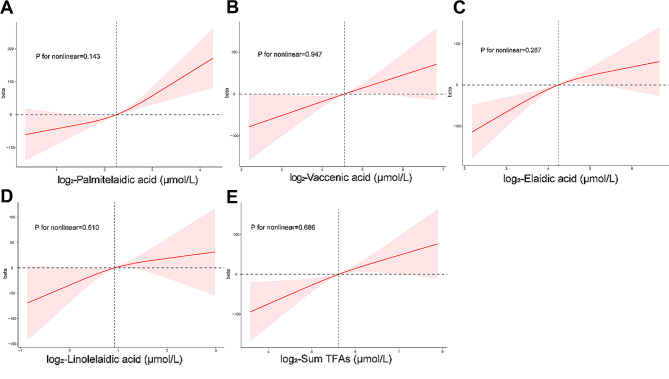
The restricted cubic splines analysis of the association between log2-Palmitelaidic acid (**A**), log2-Vaccenic acid (**B**), log2-Elaidic acid (**C**), log2-Linolelaidic acid (**D**), log2-Sum TFAs (**E**) and SII. Abbreviations: TFAs, trans fatty acids, SII, Systemic immune-inflammation index

Furthermore, the study performed the RCS analysis for four main types of TFA and the sum of TFAs which was shown in Fig. 2. Judging from the results, no significant nonlinear correlation was observed between four main types of TFAs, the sum TFAs and SII (all *P* for nonlinear > 0.05).

### Subgroup analysis

The stratified analysis was utilized to explore the potential interactive factors in the relationship between TFAs and SII. The results were shown in Tables 3, 4, 5, 6 and 7. For the circulating palmitelaidic acid, vaccenic acid, elaidic acid, and the sum TFAs, they were more pronounced in never smokers (all *P* for interaction < 0.05). Additionally, the linolelaidic acid was more positively related to the SII in individuals with lower BMI, and a history of never having smoked (*P* for interaction < 0.05).

**Table 3:** Association between palmitelaidic acid and SII in different subgroups

Palmitelaidic acid	SII	
	β (95% CI) P value	P for interaction
**Age (years)**		0.969
<60	54.77 (31.09, 78.45) <0.001	
≥60	78.69 (-19.55, 176.93) 0.110	
**Gender**		0.872
Male	45.35 (3.40, 87.30) 0.036	
Female	62.86 (33.42, 92.31) <0.001	
**BMI (kg/m**^**2**^)		0.188
<30	68.35 (28.48, 108.22) 0.002	
≥30	34.75 (-5.49, 74.99) 0.087	
**Race**		0.152
Mexican American	149.67 (41.09, 258.25) 0.011	
Non-Hispanic Black	64.08 (12.46, 115.69) 0.019	
Non-Hispanic White	37.38 (16.01, 58.74) 0.002	
Other Hispanic	76.03 (-40.47, 192.53) 0.144	
Other Race	5.94 (-151.33, 163.21) 0.886	
**Smoking Status**		0.002
Never	90.07 (56.21, 123.93) <0.001	
Former	55.98 (1.19, 110.77) 0.046	
Current	-23.23 (-92.33, 45.87) 0.491	
**CVD**		0.239
No	56.05 (30.76, 81.35) <0.001	
Yes	75.12 (-15.92, 166.16) 0.100	
**DM**		0.807
No	59.52 (35.06, 83.98) <0.001	
Yes	49.84 (-74.67, 174.34) 0.407	
**Hypertension**		0.636
No	51.11 (27.06, 75.17) <0.001	
Yes	78.07 (-4.57, 160.71) 0.063	

Adjusted for age, gender, race, PIR, education level, BMI, marital, smoking status, CVD, alcohol consumption, uric acid, eGFR, HDL, LDL, hypertension, DM, and cancer, if not stratified

Abbreviations: SII, Systemic immune-inflammation index, PIR, Poverty income ratio, BMI, Body mass index, CVD, Cardiovascular disease, eGFR, Estimated glomerular filtration rate, HDL, high-density lipoprotein, LDL, low-density lipoprotein, DM, Diabetes mellitus, CI, Confidence interval

**Table 4:** Association between Vaccenic acid and SII in different subgroups

Vaccenic acid	SII	
	β (95% CI) P value	P for interaction
**Age (years)**		0.855
<60	28.75 (11.68, 45.82) 0.002	
≥60	53.43 (-15.16, 122.02) 0.120	
**Gender**		0.942
Male	26.20 (-1.90, 54.31) 0.066	
Female	36.95 (14.58, 59.31) 0.003	
**BMI (kg/m**^**2**^)		0.195
<30	39.50 (11.50, 67.50) 0.008	
≥30	14.66 (-18.93, 48.24) 0.373	
**Race**		0.284
Mexican American	98.96 (9.81, 188.11) 0.033	
Non-Hispanic Black	42.53 (10.96, 74.10) 0.013	
Non-Hispanic White	13.05 (-8.24, 34.33) 0.215	
Other Hispanic	53.29 (-41.58, 148.17) 0.194	
Other Race	0.74 (-97.27, 98.76) 0.977	
**Smoking Status**		<0.001
Never	59.52 (35.36, 83.67) <0.001	
Former	14.50 (-20.02, 49.02) 0.390	
Current	-26.14 (-67.07, 14.78) 0.198	
**CVD**		0.177
No	29.11 (11.84, 46.38) 0.002	
Yes	45.02 (-15.56, 105.60) 0.135	
**DM**		0.845
No	32.08 (14.49, 49.68) 0.001	
Yes	14.62 (-66.62, 95.87) 0.707	
**Hypertension**		0.879
No	29.88 (12.86, 46.89) 0.002	
Yes	39.15 (-16.47, 94.77) 0.158	

Adjusted for age, gender, race, PIR, education level, BMI, marital, smoking status, CVD, alcohol consumption, uric acid, eGFR, HDL, LDL, hypertension, DM, and cancer, if not stratified

Abbreviations: SII, Systemic immune-inflammation index, PIR, Poverty income ratio, BMI, Body mass index, CVD, Cardiovascular disease, eGFR, Estimated glomerular filtration rate, HDL, high-density lipoprotein, LDL, low-density lipoprotein, DM, Diabetes mellitus, CI, Confidence interval

**Table 5:** Association between elaidic acid and SII in different subgroups

Elaidic acid	SII	
	β (95% CI) P value	P for interaction
**Age (years)**		0.633
<60	48.03 (30.26, 65.81) <0.001	
≥60	49.59 (-7.80, 106.98) 0.087	
**Gender**		0.457
Male	43.65 (18.14, 69.17) 0.002	
Female	45.47 (24.23, 66.50) <0.001	
**BMI (kg/m**^**2**^)		0.131
<30	52.45 (27.21, 77.69) <0.001	
≥30	29.05 (1.14, 56.97) 0.042	
**Race**		0.438
Mexican American	73.09 (32.69, 113.49) 0.002	
Non-Hispanic Black	40.12 (-1.04, 81.27) 0.055	
Non-Hispanic White	35.99 (16.00, 55.98) 0.001	
Other Hispanic	77.12 (-9.66, 163.90) 0.069	
Other Race	14.22 (-88.53, 116.97) 0.612	
**Smoking Status**		<0.001
Never	73.03 (51.54, 94.52) <0.001	
Former	24.93 (-12.36, 62.22) 0.178	
Current	-10.77 (-51.50, 31.35) 0.618	
**CVD**		0.405
No	45.51 (28.77,62.25) <0.001	
Yes	48.07 (-16.41, 112.54) 0.134	
**DM**		0.290
No	48.16 (30.67, 65.64) <0.001	
Yes	1.39 (-71.82, 74.59) 0.968	
**Hypertension**		0.932
No	46.52 (29.08, 63.96) <0.001	
Yes	45.37 (0.10, 90.63) 0.050	

Adjusted for age, gender, race, PIR, education level, BMI, marital, smoking status, CVD, alcohol consumption, uric acid, eGFR, HDL, LDL, hypertension, DM, and cancer, if not stratified

Abbreviations: SII, Systemic immune-inflammation index, PIR, Poverty income ratio, BMI, Body mass index, CVD, Cardiovascular disease, eGFR, Estimated glomerular filtration rate, HDL, high-density lipoprotein, LDL, low-density lipoprotein, DM, Diabetes mellitus, CI, Confidence interval

**Table 6:** Association between Linolelaidic acid and SII in different subgroups

Linolelaidic acid	SII	
	β (95% CI) P value	P for interaction
**Age (years)**		0.411
<60	40.73 (18.37, 63.09) 0.001	
≥60	38.37 (-26.74, 103.48) 0.233	
**Gender**		0.697
Male	33.59 (0.68, 66.50) 0.046	
Female	39.21 (12.70, 65,73) 0.006	
**BMI (kg/m**^**2**^)		0.010
<30	56.47 (27.86, 85.08) <0.001	
≥30	-4.94 (-41.05, 31.18) 0.778	
**Race**		0.126
Mexican American	53.51 (-28.07, 135.08) 0.177	
Non-Hispanic Black	37.00 (-19.12, 93.12) 0.175	
Non-Hispanic White	20.83 (-3.82, 45.49) 0.093	
Other Hispanic	94.53 (-9.96, 199.02) 0.066	
Other Race	-19.29 (-139.01, 100.44) 0.560	
**Smoking Status**		0.003
Never	70.76 (41.23, 100.30) <0.001	
Former	-1.14 (-44.33, 42.05) 0.957	
Current	-14.89 (-67.70, 37.93) 0.563	
**CVD**		0.529
No	37.06 (16.59, 57.53) 0.001	
Yes	25.01 (-44.59, 94.61) 0.459	
**DM**		0.185
No	41.54 (18.95, 64.13) 0.001	
Yes	-48.56 (-128.62, 31.50) 0.216	
**Hypertension**		0.403
No	41.83 (16.28, 67.39) 0.268	
Yes	28.20 (-23.43, 79.82) 0.003	

Adjusted for age, gender, race, PIR, education level, BMI, marital, smoking status, CVD, alcohol consumption, uric acid, eGFR, HDL, LDL, hypertension, DM, and cancer, if not stratified

Abbreviations: SII, Systemic immune-inflammation index, PIR, Poverty income ratio, BMI, Body mass index, CVD, Cardiovascular disease, eGFR, Estimated glomerular filtration rate, HDL, high-density lipoprotein, LDL, low-density lipoprotein, DM, Diabetes mellitus, CI, Confidence interval

**Table 7:** Association between sum TFAs and SII in different subgroups

Sum TFAs	SII	
	β (95% CI) P value	P for interaction
**Age (years)**		0.920
<60	42.41 (23.45, 61.38) <0.001	
≥60	58.61 (-13.11, 130.34) 0.104	
**Gender**		0.844
Male	37.62 (8.21, 67.03) 0.015	
Female	46.90 (23.39, 70.42) <0.001	
**BMI (kg/m**^**2**^)		0.145
<30	51.70 (22.21, 81.18) 0.002	
≥30	23.84 (-8.88, 56.56) 0.144	
**Race**		0.298
Mexican American	96.94 (33.44, 160.43) 0.006	
Non-Hispanic Black	46.22 (6.78, 85.66) 0.026	
Non-Hispanic White	27.54 (6.23, 48.85) 0.014	
Other Hispanic	73.11 (-29.19, 175.41) 0.118	
Other Race	5.46 (-100.64, 111.56) 0.845	
**Smoking Status**		<0.001
Never	73.77 (48.41, 99.13) <0.001	
Former	24.06 (-15.14, 63.26) 0.214	
Current	-20.83 (-66.09, 24.42) 0.348	
**CVD**		0.268
No	41.75 (23.21, 60.29) <0.001	
Yes	52.07 (-17.93, 122.06) 0.135	
**DM**		0.509
No	45.07 (25.81, 64.32) <0.001	
Yes	7.88 (-74.24, 89.99) 0.841	
**Hypertension**		0.972
No	42.58 (23.81, 61.36) <0.001	
Yes	47.35 (-9.12, 103.83) 0.096	

Adjusted for age, gender, race, PIR, education level, BMI, marital, smoking status, CVD, alcohol consumption, uric acid, eGFR, HDL, LDL, hypertension, DM, and cancer, if not stratified

Abbreviations: SII, Systemic immune-inflammation index, PIR, Poverty income ratio, BMI, Body mass index, CVD, Cardiovascular disease, eGFR, Estimated glomerular filtration rate, HDL, high-density lipoprotein, LDL, low-density lipoprotein, DM, Diabetes mellitus, CI, Confidence interval

## Discussion

To our knowledge, there is currently limited research investigating the association between TFAs and SII. Therefore, we employed various advanced statistical models to comprehensively evaluate the influence of TFAs on SII levels. These findings revealed a positive correlation between palmitelaidic acid, vaccenic acid, elaidic acid, the total sum of TFAs, and SII in fully adjusted models. Notably, significant interactions were observed between smoking and certain TFAs.

SII is increasingly recognized as a potential biomarker for conditions such as gastrointestinal malignancies, prostate cancer, cardiovascular illnesses, and others [25–27]. In a cross-sectional study involving 730 healthy women from the Nurses’ Health Investigation I cohort, Lopez-Garcia et al. noted a positive correlation between TFAs intake and plasma concentrations of C-reactive protein (CRP), sE-selectin, sICAM-1, tumor necrosis factor-alpha receptors 2, and sVCAM-1 [28]. These findings were consistent with other interventional and observational studies that suggest consumption of TFAs could elevate inflammatory markers in the blood such as CRP, interleukin-1β, chemokine ligand 2 and interleukin-6 (IL-6) [27, 29, 30]. Further evidence from in vitro tests and animal models shows that TFAs can activate and accumulate macrophages, as well as activate NF-κB and enhance osteopontin production in the liver [31–34].

Another possible explanation for the correlation between TFAs and SII is the reduced proportion of gram-negative sulfate-reducing bacteria after a meal high in TFAs according to Ge et al. [35]. The bacteria’s subsequent overproduction of hydrogen sulfide (H_2_S) may be a factor in inflammatory bowel disease and bowel illnesses linked to inflammation [36]. By reducing the disulfide bonds in the mucus network, H_2_S promotes the breakdown of the mucus barrier and increases the permeability of the mucus layer [37]. When the mucus barrier is breached, germs and toxins can get in intimate contact with the colonic epithelium, which can lead to inflammation [37]. Owing to these inflammatory variables, a conceivable biological process that results in greater SII is excessive consumption of TFAs with pro-inflammatory properties.

The subgroup analysis and interaction tests conducted in this study revealed a noteworthy positive correlation between total TFAs and SII within subgroups categorized by smoking status, while the similar connection between the Linolelaidic acid and SII within subgroups categorized by BMI and smoking status. According to these findings, there was a higher positive association between SII scores and TFAs among nonsmokers. Previous studies have demonstrated that inflammation is frequently involved in the pathogenesis of illnesses associated with cigarette smoking [38]. The subgroup analysis’s findings further imply that the association between SII and TFAs varies according to BMI. Patients with a BMI under 30 kg/m² showed a greater correlation between TFAs and SII. Previous studies have connected TFA intake to higher BMI levels [39]. Studies suggest that BMI, a risk factor for various cancers, is associated with an elevation in SII [40]. Collectively, these results imply that those with high amounts of circulating TFAs should be closely detected for elevated SII, especially those without harmful lifestyle choices, which was consistent with previous findings [41, 42]. Nevertheless, additional investigations are necessary to clarify the specific mechanisms involved.

## Strengths and limitations

The research offers some fresh perspectives in this area. First, the study assessed the connection between TFAs and SII in U.S. adults for the first time. In addition, subgroup analyses were carried out to guarantee consistent results, and a wide range of potential confounding factors were taken into account in this study. Furthermore, after controlling for a wide range of potential confounders, the study discovered that the dose-response correlations of SII with all types of TFAs level and the sum TFAs were not nonlinear. However, some limitations of the investigation must be acknowledged. Initially, due to regulatory modifications in the past decade, the findings derived from data collected between 1999 and 2000 and 2009–2010 may not precisely depict the present scenario of TFAs intake among adults in the US. Furthermore, the results could not suggest the habits of the diet and lifestyle and the level of circulating trans fatty acids in the current Americans. Nevertheless, these results could establish a foundational reference point for subsequent analyses, given that they are grounded in the most recent data accessible for the entire adult US population. Second, even though the research employed the blood cell count-based comprehensive index as a biomarker of systemic immune inflammation, more research is necessary to determine the relationship between TFAs exposure and other biomarkers including CRP and IL-6. Thirdly, given the cross-sectional study design employed, the investigation is unable to establish causation from these findings. Consequently, even though variables were taken into account, measurement errors and uncontrolled confounders might have had an impact on the results.

## Conclusion

In this cross-sectional study, the circulating TFAs were investigated to be positively associated with SII, and a nonlinear relationship was found. Notably, these associations could be more weakened or more pronounced in different subgroups. Briefly, the findings of the study emphasize the potential role of TFAs in systemic inflammation severity and provide new insights into controlling systemic inflammation levels in the US general population from a dietary health perspective. Nevertheless, additional research is essential to explore the cause-and-effect relationship and to elucidate the specific underlying mechanism.

### Electronic supplementary material

Below is the link to the electronic supplementary material.

Supplementary Material 1

## Data Availability

The study utilized data from the National Health and Nutrition Examination Survey (NHANES), which is publicly available in the NHANES repository, https://www.cdc.gov/nchs/nhanes.
