# Supplementary material for: Associations between trans fatty acids and systemic immune-inflammation index: a cross-sectional study
Source: Lipids Health Dis. 2024 Apr 27;23:122. doi: 10.1186/s12944-024-02109-w (PMC11055356; doi:10.1186/s12944-024-02109-w)
Supplement: Supplementary file 1 — Supplementary Material 1 [file 12944_2024_2109_MOESM1_ESM.pdf]

Associations_between_trans_fatty_acids_and_systemic_immune_inflammation_index_a_cross_sectional_study

WORD COUNT 3078
TIME SUBMITTED 13-APR-2024 11:46AM
PAPER ID 108236381

Abstract

Background:

Previous studies have demonstrated that trans fatty acids (TFAs) intake was linked to an increased risk of chronic diseases. As a novel systemic inflammatory biomarker, the clinical value and efficacy of the systemic immune-inflammation index (SII) have been widely explored. However, the association between TFAs and SII is still unclear. Therefore, the study aims to investigate the connection between TFAs and SII in US adults.

Methods:

The study retrieved data from the National Health and Nutrition Examination Survey (NHANES) for the years 1999-2000 and 2009-2010. Following the exclusion of ineligible participants, the study encompassed a total of 3047 individuals. The research employed a multivariate linear regression model to investigate the connection between circulating TFAs and SII. Furthermore, the restricted cubic spline (RCS) model was utilized to evaluate the potential nonlinear association. Subgroup analysis was also conducted to investigate the latent interactive factors.

Results:

In this investigation, participants exhibited a mean age of 47.40 years, with 53.91% of them being female. Utilizing a multivariate linear regression model, the independent positive associations between the log2-transformed palmitelaidic acid, the log2 transformed-vaccenic acid, the log2-transformed elaidic acid, the log2-transformed linolelaidic acid, and the log2-transformed-total sum of TFAs with the SII (all $P<0.05$) were noted. In the RCS analysis, no nonlinear relationship was observed between the log2-transformed palmitelaidic acid, the log2 transformed-vaccenic acid, the log2-transformed elaidic acid, the log2-transformed linolelaidic acid, the log2-transformed-total sum of TFAs and the SII (all P for nonlinear>0.05). For the stratified analysis, the relationship between the circulating TFAs and the SII differed by the obesity status and the smoking status.

Conclusions:

A positive association was investigated between three types of TFA, the sum of TFAs, and the SII in the US population. Additional rigorously designed studies are needed to verify

the results and explore the potential mechanism.

1. Introduction

Trans fatty acids (TFAs) are a specific type of unsaturated acids that are naturally occurring and artificially produced. In the U.S., dietary TFAs account for 2-3% of the energy intake, primarily from processed foods, including baked products and packaged snacks[1]. However, TFAs are not essential to the human body and are detrimental to health. Earlier investigations have established that the intake of TFAs is associated with an increase in lipid levels[2, 3], which may lead to an increased prevalence of cardiovascular diseases[4]. Moreover, studies based on in vivo and in vitro models found that the TFAs could not only modulate the microbiome in the mice but also induce inflammation and oxidative stress[5, 6], which are associated with the risk of some common chronic diseases[7].

It has been proposed that inflammation is a major factor in the development of diseases. To better evaluate the systematic inflammation of patients in clinical practice, a novel blood inflammation biomarker called the systematic immune-inflammation index (SII) has been proposed, which could be calculated based on three types of blood cells (lymphocytes, neutrophils, and platelets)[8]. As an easily accessible indicator, plenty of studies have investigated and confirmed its prognostic value in diabetes, lung cancer, and the general population[9-11]. A study based on 6003 Chinese adults discovered that the SII was significantly associated with hypertension over a long-term period[12]. In addition, recent studies have found that elevated SII may increase the risk of diabetic retinopathy and cognitive impairment, as well as the severity of carotid artery stenosis[13-15].

Some studies have reported that a few dietary factors, including dietary fiber, vitamin D and selenium, may influence systemic inflammation in humans[16-18]. However, information on the association between TFAs and systemic inflammation is limited. Given the widespread use of TFAs and the excellent efficacy of SII, exploring the relationship between circulating TFAs and SII may provide some novel insights into the adverse effects of TFAs on inflammation. Hence, National Health and Nutrition Examination Survey (NHANES) data collected during the years 1999–2000 and 2009–2010 were used in the study to explore

the connections between plasma TFAs and SII among U.S. adults.

2. Methods

2.1 Study population

NHANES is a large database that could be freely accessed by researchers around the globe. The Centers for Disease Control and Prevention (CDC) conducted the NHANES project on a two-year cycle to evaluate the nutritional and medical status of non-institutionalized individuals living in the U.S. Approximately 5000 civilians living in the communities were selected by authorities across each cycle. The complex sampling and multi-stage methodology was utilized in the sample survey to generate nationally representative data.

The research selected participants' data from two survey cycles of the database (1999-2000 and 2009-2010), for which the level of circulating TFAs was available. In this study, a total of 20502 participants aged ≥ 20 years were first extracted. Then, we excluded 13642 samples with missing data on TFAs in the second step and 29 samples with missing data on SII in the third step. Furthermore, 3784 participants with missing data on the covariates were also regarded as ineligible. Finally, 3047 eligible U.S. adults from the NHANES were included to conduct a cross-sectional study. The flowchart of the inclusion and exclusion criteria is shown in Figure 1. The protocol was approved by the Ethical Review Committee of the National Health Council, and each individual gave written informed consent.

2.2 Measurement of circulating TFA

Previous studies have reported detailed methods and approaches to evaluate the level of plasma TFA[19, 20]. In brief, participants' blood samples were obtained in the morning after a fasting period following the protocol outlined by the CDC. Subsequently, TFA isomers were identified by their chromatographic retention times and specific mass-to-charge ratios. Quantification of metabolites was conducted using established standard solutions, incorporating stable isotope-labeled fatty acids as internal standards. The total amount of TFAs was determined as follows: Sum TFAs = vaccenic acid + linoelaidic acid + palmitelaidic acid + elaidic acid.

2.3 Identification of SII

The study derived the SII by multiplying the number of neutrophils by the number of platelets, followed by dividing by the number of lymphocytes. The level of the complete blood cell count is expressed as ×103 cells/μl and was assessed by blood analysis equipment, which is conducted by professional laboratory staff.

2.4 Covariates

Considering the clinical facts, the potential confounding factors were included in the study. Demographic factors, including age, gender, race, education, poverty income ratio (PIR), and marital status, were evaluated through a questionnaire conducted at the mobile examination center. Race was categorized into five groups: Mexican American, non-Hispanic Black, non-Hispanic White, other Hispanic, and other races. Marital status was categorized as married/living with a partner, widowed/divorced/separated, or never married. Smoking status was defined based on lifetime cigarette consumption, with categories for never smoked, ever smoked, and current smoker. Alcohol consumption was determined by the mean alcohol intake over a two-day diet obtained through dietary recall. Education level was stratified into three groups: less than high school, high school graduate, and more than high school. Trained medical personnel measured and calculated participants' body mass index (BMI) during interviews. Information on cardiovascular disease (CVD), hypertension, cancer, and diabetes mellitus (DM) was collected through questionnaires. Specifically speaking, participants were considered CVD patients, based on the previous studies[21-23]. The direct immunoassay-related equipment was utilized for examining the level of the lipids in individuals. Serum uric acid levels were measured using the colorimetric method in laboratory tests, and the estimated glomerular filtration rate (eGFR) was calculated following established research protocols.[24].

2.5 Statistical analysis

Based on the CDC guideline, all analyses involved in the study took clustering, multi-stage, and sample weights into consideration. Given the skewed distribution of TFAs, a log2 transformation was applied for the regression analysis. The baseline characteristics of participants were stratified by the tertiles of sum TFAs. Continuous variables were presented as mean ± standard error using weighted linear regression models, while categorical

variables were expressed as percentages through the Rao-Scott chi-square test. Subsequently, the research employed the multivariate linear regression model to examine the relationship between TFAs and SII. The effect size (β) and 95% confidence intervals (CI) were calculated for statistical assessment. Model 1 was unadjusted, while Model 2 accounted for age, gender, and race. Model 3 was adjusted for the all latent confounders we included for the present investigation to verify the robustness of the results. Additionally, the restricted cubic spline (RCS) model was utilized to investigate potential non-linear associations involving four main types of TFAs, the sum TFAs, and SII. Furthermore, subgroup analysis and interactive *P* values were utilized to probe potential interaction effects among stratified variables. All analyses were conducted using R software (version 4.2.1).

3. Results

3.1 Baseline characteristics of the study participants

Table 1 presents the weighted basic characteristics of 3047 individuals. In the study population, the average age was 47.40 years, and 53.91% were female. Additionally, the mean levels of the circulating palmitelaidic acid, vaccenic acid, elaidic acid and linolelaidic acid were 5.05 μmol/L, 25.87 μmol/L, 20.99 μmol/L, and2.07 μmol/L, respectively. After classifying by sum TFAs tertiles, individuals with higher circulating TFAs were more likely to be older, non-Hispanic White, have lower educational attainment, married/living with a partner, current smokers, less alcohol consumption, lower eGFR, and higher SII. However, no statistically significant difference was shown in gender, PIR, uric acid, CVD, hypertension, DM, and cancer across the three groups. Interestingly, BMI was shown to be highest in the T2 group with an average of 29.24kg/m2 and the population in the T2 group had the highest age with an average of 48.49 years.

3.2 Relationship between TFAs and SII

The multivariate linear regression model was performed and detailed results were shown in Table 2. In the crude model (model 1), the four types of TFA and the sum of TFAs were significantly and positively related to SII. After adjusting for age, sex, and race (model 2), the relationship was weakened. After adjusting for the covariates that were included in the study in Model 3, the connection between the log2-transformed palmitelaidic acid β=56.84, 95% CI=30.93, 82.74, $P<0.001$), the log2-transformed vaccenic acid β=32.28, 95% CI=14.99,

49.57, $P=0.002$), the log2-transformed elaidic acid $\beta=40.31$, 95% CI=23.09, 57.54, $P<0.001$), the log2-transformed-linolelaidic acid $\beta=27.04$, 95% CI=6.10, 47.97, $P=0.016$), the log2-transformed sum TFAs ($\beta=40.33$, 95% CI=21.29, 59.38, $P<0.001$) and SII remain robust. Compared to the T1 group, individuals in the T3 group of palmitelaidic acid ($\beta=75.19$, 95% CI=25.38, 125.00, $P=0.007$), vaccenic acid ($\beta=62.02$, 95% CI=11.02, 113.02, $P=0.022$), elaidic acid $\beta=84.43$, 95% CI=34.80, 134.07, $P=0.003$), and sum TFAs $\beta=78.08$, 95% CI=31.74, 124.41, $P=0.003$) were significantly had higher SII. However, the population in the T3 group of the linolelaidic acid was not observed to have a higher SII ($P>0.05$).

Furthermore, the study performed the RCS analysis for four main types of TFA and the sum of TFAs which was shown in Figure 2. Judging from the results, no significant nonlinear correlation was observed between four main types of TFAs, the sum TFAs and SII (all P for nonlinear>0.05).

3.3 Subgroup analysis

The stratified analysis was utilized to explore the potential interactive factors in the relationship between TFAs and SII. The results were shown in Table 3-7. For the circulating palmitelaidic acid, vaccenic acid, elaidic acid, and the sum TFAs, they were more pronounced in never smokers (all P for interaction <0.05). Additionally, the linolelaidic acid was more positively related to the SII in individuals with lower BMI, and a history of never having smoked (P for interaction <0.05).

4. Discussion

To our knowledge, there is currently limited research investigating the association between TFAs and SII. Therefore, we employed various advanced statistical models to comprehensively evaluate the influence of TFAs on SII levels. These findings revealed a positive correlation between palmitelaidic acid, vaccenic acid, elaidic acid, the total sum of TFAs, and SII in fully adjusted models. Notably, significant interactions were observed between smoking and certain TFAs.

SII is increasingly recognized as a potential biomarker for conditions such as gastrointestinal malignancies, prostate cancer, cardiovascular illnesses, and others[25-27]. In a cross-sectional study involving 730 healthy women from the Nurses' Health Investigation I

cohort, Lopez-Garcia et al. noted a positive correlation between TFAs intake and plasma concentrations of C-reactive protein (CRP), sE-selectin, sICAM-1, tumor necrosis factor-alpha receptors 2, and sVCAM-1[28]. These findings were consistent with other interventional and observational studies that suggest consumption of TFAs could elevate inflammatory markers in the blood such as CRP, interleukin-1β, chemokine ligand 2 and interleukin-6 (IL-6)[27, 29, 30]. Further evidence from in vitro tests and animal models shows that TFAs can activate and accumulate macrophages, as well as activate NF-κB and enhance osteopontin production in the liver[31-34].

Another possible explanation for the correlation between TFAs and SII is the reduced proportion of gram-negative sulfate-reducing bacteria after a meal high in TFAs according to Ge et al.[35]. The bacteria's subsequent overproduction of hydrogen sulfide (H_2S) may be a factor in inflammatory bowel disease and bowel illnesses linked to inflammation[36]. By reducing the disulfide bonds in the mucus network, H_2S promotes the breakdown of the mucus barrier and increases the permeability of the mucus layer[37]. When the mucus barrier is breached, germs and toxins can get in intimate contact with the colonic epithelium, which can lead to inflammation[37]. Owing to these inflammatory variables, a conceivable biological process that results in greater SII is excessive consumption of TFAs with pro-inflammatory properties.

The subgroup analysis and interaction tests conducted in this study revealed a noteworthy positive correlation between total TFAs and SII within subgroups categorized by smoking status, while the similar connection between the Linolelaidic acid and SII within subgroups categorized by BMI and smoking status. According to these findings, there was a higher positive association between SII scores and TFAs among nonsmokers. Previous studies have demonstrated that inflammation is frequently involved in the pathogenesis of illnesses associated with cigarette smoking[38]. The subgroup analysis's findings further imply that the association between SII and TFAs varies according to BMI. Patients with a BMI under 30 kg/m^2 showed a greater correlation between TFAs and SII. Previous studies have connected TFA intake to higher BMI levels[39]. Studies suggest that BMI, a risk factor for various cancers, is associated with an elevation in SII[40]. Collectively, these results imply that those with high amounts of circulating TFAs should be closely detected for elevated SII,

especially those without harmful lifestyle choices, which was consistent with previous findings[41, 42]. Nevertheless, additional investigations are necessary to clarify the specific mechanisms involved.

The research offers some fresh perspectives in this area. First, the study assessed the connection between TFAs and SII in U.S. adults for the first time. In addition, subgroup analyses were carried out to guarantee consistent results, and a wide range of potential confounding factors were taken into account in this study. Furthermore, after controlling for a wide range of potential confounders, the study discovered that the dose-response correlations of SII with all types of TFAs level and the sum TFAs were not nonlinear.

5. Strengths and limitations

However, some limitations of the investigation must be acknowledged. Initially, due to regulatory modifications in the past decade, the findings derived from data collected between 1999-2000 and 2009-2010 may not precisely depict the present scenario of TFAs intake among adults in the US. Furthermore, the results could not suggest the habits of the diet and lifestyle and the level of circulating trans fatty acids in the current Americans. Nevertheless, these results could establish a foundational reference point for subsequent analyses, given that they are grounded in the most recent data accessible for the entire adult US population. Second, even though the research employed the blood cell count-based comprehensive index as a biomarker of systemic immune inflammation, more research is necessary to determine the relationship between TFAs exposure and other biomarkers including CRP and IL-6. Thirdly, given the cross-sectional study design employed, the investigation is unable to establish causation from these findings. Consequently, even though variables were taken into account, measurement errors and uncontrolled confounders might have had an impact on the results.

6. Conclusion

In this cross-sectional study, the circulating TFAs were investigated to be positively associated with SII, and a nonlinear relationship was found. Notably, these associations could be more weakened or more pronounced in different subgroups. Briefly, the findings of the

study emphasize the potential role of TFAs in systemic inflammation severity and provide new insights into controlling systemic inflammation levels in the US general population from a dietary health perspective. Nevertheless, additional research is essential to explore the cause-and-effect relationship and to elucidate the specific underlying mechanism.

Associations_between_trans_fatty_acids_and_systemic_im...

ORIGINALITY REPORT

9%
SIMILARITY INDEX

PRIMARY SOURCES

1	www.frontiersin.org<br>Internet	117 words — 4%
2	www.mdpi.com<br>Internet	31 words — 1%
3	www.ncbi.nlm.nih.gov<br>Internet	28 words — 1%
4	Xiaoqian Wang, Fengjuan Jiang, Wenqing Chen, Hui Yuan, Yuan Li. "The Association Between Circulating Trans Fatty Acids and Thyroid Function Measures in U.S. Adults", Frontiers in Endocrinology, 2022<br>Crossref	21 words — 1%
5	assets.researchsquare.com<br>Internet	19 words — 1%
6	bmcnephrol.biomedcentral.com<br>Internet	16 words — 1%
7	Jiancun Wang, Qiang Xue, Xuewen Tan, Jie Huang, Yibai Zhu, Wen Li. "Effects of light perception on visual function recovery in patients with traumatic optic neuropathy", Research Square Platform LLC, 2023<br>Crossref Posted Content	14 words — < 1%

8	www.cambridge.org<br>Internet	13 words — < 1%
9	doaj.org<br>Internet	9 words — < 1%
10	www.nlsinfo.org<br>Internet	9 words — < 1%
11	translational-medicine.biomedcentral.com<br>Internet	8 words — < 1%
12	www.techxprtz.com<br>Internet	8 words — < 1%

EXCLUDE QUOTES	ON	EXCLUDE SOURCES	OFF
EXCLUDE BIBLIOGRAPHY	ON	EXCLUDE MATCHES	OFF
